# News and Events of the Week

**Published:** 1910-05-07

**Authors:** 


### News and Events of the Week.

Dr. Norman Moore has been elected Librarian of the Royal College of Physicians.

The Bedford College for Women has been added to the list of institutions recognised by the Royal College of Physicians.

Among the new Bills brought in and read a first time last week was Sir W. Collins' (St. Pancras, W., Min.), Bill to amend the law relating to the certification and registration of deaths and burials.

A portrait of Sir Henry Morris, by W. W. Ouless, R.A., is hung on the line in the principal room at this year's exhibition of the Royal Academy at Burlington House. This is the chief picture of strictly medical interest in the galleries. In the genre pictures the doctor is this time conspicuous by his absence.

A recent decision of the board of the Leeds Infirmary admits women students in future to the practice of the infirmary. As the lectures and laboratory courses of the medical school of the University are already open to women, this decision will allow them to receive the whole of their medical education at Leeds.

The next award of the Royal College of Physicians, under the foundation of the Weber Parkes Prize and Medals, will be made in 1912, and the adjudicators have selected as the subject of the essay "The influence of mixed and secondary infections upon pulmonary tuberculosis in man, and the measures preventive and curative for dealing with them."

Last week's *Gazette* contains an Order in Council signifying his Majesty's pleasure, with the advice of the Privy Council, in pursuance of the provisions of the Medical Acts, 1858 and 1886, to nominate Dr. Arthur Newsholme, M.D., M.R.C.S., to be, for five years, a member of the General Council of Medical Education and Registration of the United Kingdom, as from May 15, 1910.

Dr. F. W. Andrewes will deliver the Croonian Lectures in June. The Harveian Oration will be delivered by Dr. H. B. Donkin on St. Luke's Day, October 18; the Bradshaw Lecture by Dr. G. N. Pitt; the FitzPatrick Lectures, on "The History of Medicine," by Sir T. Clifford Allbutt; and the Horace Dobell Lecture, by Dr. W. Bulloch, will be delivered in November.

Miss Fay Lankester, hon. secretary of the National Health Society, 53 Berners Street, Oxford Street, in a recent letter to the *Times*, said: "I am writing on behalf of the Committee of the National Health Society to beg the public to co-operate with the Society in forming a lectureship to be a memorial of the late Lady Priestley, who was one of its promoters. The Committee is anxious to secure the services and eloquence of the most eminent authorities to give annual lectures on whatever subject may be most important for the moment relating to hygiene or sanitation. The Committee has already received much encouragement, but the sum of £300, the desired amount, has not yet been reached, and it is for this purpose that we appeal to the public and perhaps to friends of the Society who may not yet have had notice of the intentions of the Committee."

Under the title of the *Journal of Genetics*, it is proposed to publish in or about next August a periodical for original research in heredity, variation, and allied subjects. It will also, from time to time, contain articles summarising the existing state of knowledge in the various branches of genetics, but reviews and abstracts of work published elsewhere, as a rule, will not be included. Illustration will be provided, and, where the subject-matter demands it, illustration in colour. Communications should be made to Professor Punnett, Caius College, Cambridge.

The annual report of the Brighton medical officer of health (Dr. Duncan Forbes) states that during 1909 the infantile mortality rate was 95 per 1,000 births registered, the lowest on record for Brighton. For the previous ten years the average was 130. The death-rate, 15.25 per 1,000, was slightly higher than that for England and Wales as a whole, 14.5. The conclusion to be drawn from these facts, says the medical officer, is that a portion of the Brighton death-rate is due to its high reputation as a health resort.

The Essex Education Committee has been discussing recently a proposal for the medical inspection of new entrants to secondary schools. A special committee recommended that arrangements should be made for the inspection of boys by one of the county medical inspectors, and that for the inspection of girls a lady doctor should be specially appointed. It has been decided to refer the proposal to the governing bodies of the secondary schools and to local education authorities for their opinion.

At the quarterly meeting of the Royal College of Physicians of London last week a letter was received from the trustees of London Parochial Charities concerning a new scheme for the management of the Chelsea Physic Garden, and the College resolved: (*a*) "That the altered scheme of the Charity Commissioners in respect to the Chelsea Physic Garden be approved, and its provisions given effect to, so far as they relate to this College; (*b*) that the College make a donation of ten guineas to the funds of the Chelsea Physic Garden."

A meeting of the members of the Psycho-Therapeutic Society, the object of which is "the study, investigation, and practice of health reform, medical hypnotism, suggestive therapeutics, curative human radiations, and drugless healing, with due regard to diet, hygiene, and the observance of natural laws of health," was held last Saturday at 34 Bloomsbury Square, to inaugurate their new headquarters. Mr. George Spriggs, President, occupied the chair, and among those present were Dr. Robert Bell, Dr. Forbes Winslow, Dr. J. Stenson Hooker, and Mr. Arthur Hallam (hon. secretary). The President stated that during the nine years of the Society's existence it had successfully attended about 2,500 patients, and given 22,000 free treatments. Dr. Forbes Winslow said that more than two-thirds of every form of nervous or mental disease could be treated by psycho-therapeutics. He proposed shortly to hold a meeting in London on the question of lunacy law reform, contending that lunatic asylums were not proper for temporary cases that could be cured by suggestion and were sometimes incarcerated by interested friends.

